# Supplementary material for: Digital physiotherapy assessment vs conventional face-to-face physiotherapy assessment of patients with musculoskeletal disorders: A systematic review
Source: PLoS One. 2023 Mar 21;18(3):e0283013. doi: 10.1371/journal.pone.0283013 (PMC10030027; doi:10.1371/journal.pone.0283013)
Supplement: S2 File — (DOCX) [file pone.0283013.s002.docx]

**Supporting file 2** Search strategies and results

**Databases searched:** Medline (OvidSP), Cochrane Library, Cinahl (EBSCO), AMED (EBSCO), PEDro

**Database:** Ovid MEDLINE(R) ALL

**Date:** 26 May 2021

**No. of results:** 770 ref

| **#** | **Searches** | **Results** |
| --- | --- | --- |
| 1 | exp Musculoskeletal Diseases/ | 1115888 |
| 2 | ((musculoskelet* or orthopedic* or ortopedic* or orthopaed* or ortopaed* or musc* or skelet* or bone* or cartilag* or joint* or shoulder* or elbow* or wrist* or neck* or cervical or back* or spine or spinal or lumb* or hip* or coxa* or knee* or ankle* or tars* or foot or feet) adj3 (disease* or condition* or disorder* or problem* or pain* or ache* or injur* or damage* or diagnos* or complaint* or dysfunction*)).ab,kf,ti. | 529904 |
| 3 | (backpain* or backache* or lumbago or dorsalgia or arthralgia* or polyarthralgia* or neckpain* or neckache* or cervicalgia* or cervicodynia*).ab,kf,ti. | 15104 |
| 4 | 1 or 2 or 3 | 1490798 |
| 5 | exp Telemedicine/ or exp Videoconferencing/ | 35758 |
| 6 | (assess* or examin* or measur* or evaluat* or test* or diagnos* or estimat* or judg* or visit* or consult* or counsel* or appointment*).ab,kf,ti. | 13179598 |
| 7 | 5 and 6 | 21700 |
| 8 | ((digital* or electronic* or virtual or interactive* or inter-activ* or tele* or ehealth* or e-health* or mhealth* or m-health* or app or apps or smartphone* or phone* or video* or online or internet* or webbased or web-based or computer*) adj6 (assess* or examin* or measur* or evaluat* or test* or diagnos* or estimat* or judg* or visit* or consult* or counsel* or appointment*)).ab,kf,ti. | 222320 |
| 9 | 7 or 8 | 232649 |
| 10 | exp Physical Therapists/ or exp Physical Therapy Modalities/ | 161829 |
| 11 | (physical therap* or physiotherap* or physio-therap*).ab,kf,ti. | 53764 |
| 12 | 10 or 11 | 191773 |
| 13 | 4 and 9 and 12 | 770 |

* ab = abstract, kf = author keyword, ti = title

**Database:** The Cochrane Library
**Date:** 26 May 2021
**No. of results:** 316 ref

*Cochrane reviews: 12
Cochrane protocols: 0
Trials: 304
Editorials: 0
Special collections: 0
Clinical answers: 0*

| **ID** | **Search** | **Hits** |
| --- | --- | --- |
| #1 | MeSH descriptor: [Musculoskeletal Diseases] explode all trees | 42086 |
| #2 | ((musculoskelet* OR orthopedic* OR ortopedic* OR orthopaed* OR ortopaed* OR musc* OR skelet* OR bone* OR cartilag* OR joint* OR shoulder* OR elbow* OR wrist* OR neck* OR cervical OR back* OR spine OR spinal OR lumb* OR hip* OR coxa* OR knee* OR ankle* OR tars* OR foot OR feet) NEAR/3 (disease* OR condition* OR disorder* OR problem* OR pain* OR ache* OR injur* OR damage* OR diagnos* OR complaint* OR dysfunction*)):ti,ab,kw (Word variations have been searched) | 90331 |
| #3 | (backpain* OR backache* OR lumbago OR dorsalgia OR arthralgia* OR polyarthralgia* OR neckpain* OR neckache* OR cervicalgia* OR cervicodynia*):ti,ab,kw (Word variations have been searched) | 8806 |
| #4 | #1 OR #2 OR #3 | 120129 |
| #5 | MeSH descriptor: [Telemedicine] explode all trees | 2764 |
| #6 | MeSH descriptor: [Videoconferencing] explode all trees | 216 |
| #7 | #5 OR #6 | 2862 |
| #8 | (assess* OR examin* OR measur* OR evaluat* OR test* OR diagnos* OR estimat* OR judg* OR visit* OR consult* OR counsel* OR appointment*):ti,ab,kw (Word variations have been searched) | 1222055 |
| #9 | #7 AND #8 | 2736 |
| #10 | ((digital* OR electronic* OR virtual OR interactive* OR "inter activ*" OR tele* OR ehealth* OR "e health*" OR mhealth* OR "m health*" OR app OR apps OR smartphone* OR phone* OR video* OR online OR internet* OR webbased OR "web based" OR computer* ) NEAR/6 (assess* OR examin* OR measur* OR evaluat* OR test* OR diagnos* OR estimat* OR judg* OR visit* OR consult* OR counsel* OR appointment*)):ti,ab,kw (Word variations have been searched) | 46852 |
| #11 | #9 OR #10 | 48191 |
| #12 | MeSH descriptor: [Physical Therapists] explode all trees | 121 |
| #13 | MeSH descriptor: [Physical Therapy Modalities] explode all trees | 26453 |
| #14 | ("physical therap*" OR physiotherap* OR "physio therap*"):ti,ab,kw (Word variations have been searched) | 16658 |
| #15 | #12 OR #13 OR #14 | 40199 |
| #16 | #4 AND #11 AND #15 | 537 |
| #17 | (clinicaltrials or trialsearch):so | 364015 |
| #18 | #16 NOT #17 | 373 |
| #19 | (conference abstract):pt | 173599 |
| **#20** | **#18 NOT #19** | **316** |

* ab = abstract, kw = author keyword, pt = publication type, so = source, ti = title

**Database:** Cinahl (EBSCO)
**Date:** 26 May 2021
**No. of results:** 435 ref

| **#** | **Query** | **Results** |
| --- | --- | --- |
| **S13** | **S4 AND S9 AND S12** | **435** |
| S12 | S10 OR S11 | 65,184 |
| S11 | AB ( physical therap* OR physiotherap* OR physio-therap* ) OR TI ( physical therap* OR physiotherap* OR physio-therap* ) | 63,900 |
| S10 | (MH "Physical Therapy Assessment") | 2,330 |
| S9 | S7 OR S8 | 87,220 |
| S8 | AB ( (digital* OR electronic* OR virtual OR interactive* OR inter-activ* OR tele* OR ehealth* OR e-health* OR mhealth* OR m-health* OR app OR apps OR smartphone* OR phone* OR video* OR online OR internet* OR webbased OR web-based OR computer*) N6 (assess* OR examin* OR measur* OR evaluat* OR test* OR diagnos* OR estimat* OR judg* OR visit* OR consult* OR counsel* OR appointment*) ) OR TI ( (digital* OR electronic* OR virtual OR interactive* OR inter-activ* OR tele* OR ehealth* OR e-health* OR mhealth* OR m-health* OR app OR apps OR smartphone* OR phone* OR video* OR online OR internet* OR webbased OR web-based OR computer*) N6 (assess* OR examin* OR measur* OR evaluat* OR test* OR diagnos* OR estimat* OR judg* OR visit* OR consult* OR counsel* OR appointment*) ) | 83,332 |
| S7 | S5 AND S6 | 8,863 |
| S6 | AB ( assess* OR examin* OR measur* OR evaluat* OR test* OR diagnos* OR estimat* OR judg* OR visit* OR consult* OR counsel* OR appointment* ) OR TI ( assess* OR examin* OR measur* OR evaluat* OR test* OR diagnos* OR estimat* OR judg* OR visit* OR consult* OR counsel* OR appointment* ) | 2,667,412 |
| S5 | (MH "Telemedicine+") OR (MH "Videoconferencing+") | 19,314 |
| S4 | S1 OR S2 OR S3 | 416,806 |
| S3 | AB ( backpain* OR backache* OR lumbago OR dorsalgia OR arthralgia* OR polyarthralgia* OR neckpain* OR neckache* OR cervicalgia* OR cervicodynia* ) OR TI ( backpain* OR backache* OR lumbago OR dorsalgia OR arthralgia* OR polyarthralgia* OR neckpain* OR neckache* OR cervicalgia* OR cervicodynia* ) | 2,717 |
| S2 | AB ( (musculoskelet* OR orthopedic* OR ortopedic* OR orthopaed* OR ortopaed* OR musc* OR skelet* OR bone* OR cartilag* OR joint* OR shoulder* OR elbow* OR wrist* OR neck* OR cervical OR back* OR spine OR spinal OR lumb* OR hip* OR coxa* OR knee* OR ankle* OR tars* OR foot OR feet) N3 (disease* OR condition* OR disorder* OR problem* OR pain* OR ache* OR injur* OR damage* OR diagnos* OR complaint* OR dysfunction*) ) OR TI ( (musculoskelet* OR orthopedic* OR ortopedic* OR orthopaed* OR ortopaed* OR musc* OR skelet* OR bone* OR cartilag* OR joint* OR shoulder* OR elbow* OR wrist* OR neck* OR cervical OR back* OR spine OR spinal OR lumb* OR hip* OR coxa* OR knee* OR ankle* OR tars* OR foot OR feet) N3 (disease* OR condition* OR disorder* OR problem* OR pain* OR ache* OR injur* OR damage* OR diagnos* OR complaint* OR dysfunction*) ) | 192,389 |
| S1 | (MH "Musculoskeletal Diseases+") | 278,360 |

* AB = abstract, MH = CINAHL subject heading, TI = title

**Database:** AMED (EBSCO)
**Date:** 26 May 2021
**No. of results:** 435 ref

| **#** | **Query** | **Results** |
| --- | --- | --- |
| **S6** | **S3 AND S4 AND S5** | **63** |
| S5 | AB (physical therap* OR physiotherap* OR physio-therap*) OR TI (physical therap* OR physiotherap* OR physio-therap*) | 15,366 |
| S4 | AB ((digital* OR electronic* OR virtual OR interactive* OR inter-activ* OR tele* OR ehealth* OR e-health* OR mhealth* OR m-health* OR app OR apps OR smartphone* OR phone* OR video* OR online OR internet* OR webbased OR web-based OR computer*) N6 (assess* OR examin* OR measur* OR evaluat* OR test* OR diagnos* OR estimat* OR judg* OR visit* OR consult* OR counsel* OR appointment*) ) OR TI ( (digital* OR electronic* OR virtual OR interactive* OR inter-activ* OR tele* OR ehealth* OR e-health* OR mhealth* OR m-health* OR app OR apps OR smartphone* OR phone* OR video* OR online OR internet* OR webbased OR web-based OR computer*) N6 (assess* OR examin* OR measur* OR evaluat* OR test* OR diagnos* OR estimat* OR judg* OR visit* OR consult* OR counsel* OR appointment*)) | 4,172 |
| S3 | S1 OR S2 | 28,520 |
| S2 | AB (backpain* OR backache* OR lumbago OR dorsalgia OR arthralgia* OR polyarthralgia* OR neckpain* OR neckache* OR cervicalgia* OR cervicodynia*) OR TI (backpain* OR backache* OR lumbago OR dorsalgia OR arthralgia* OR polyarthralgia* OR neckpain* OR neckache* OR cervicalgia* OR cervicodynia*) | 250 |
| S1 | AB ((musculoskelet* OR orthopedic* OR ortopedic* OR orthopaed* OR ortopaed* OR musc* OR skelet* OR bone* OR cartilag* OR joint* OR shoulder* OR elbow* OR wrist* OR neck* OR cervical OR back* OR spine OR spinal OR lumb* OR hip* OR coxa* OR knee* OR ankle* OR tars* OR foot OR feet) N3 (disease* OR condition* OR disorder* OR problem* OR pain* OR ache* OR injur* OR damage* OR diagnos* OR complaint* OR dysfunction*) ) OR TI ( (musculoskelet* OR orthopedic* OR ortopedic* OR orthopaed* OR ortopaed* OR musc* OR skelet* OR bone* OR cartilag* OR joint* OR shoulder* OR elbow* OR wrist* OR neck* OR cervical OR back* OR spine OR spinal OR lumb* OR hip* OR coxa* OR knee* OR ankle* OR tars* OR foot OR feet) N3 (disease* OR condition* OR disorder* OR problem* OR pain* OR ache* OR injur* OR damage* OR diagnos* OR complaint* OR dysfunction*)) | 28,385 |

* AB = abstract, TI = title

**Database:** PEDro
**Date:** 26 May 2021
**No. of results:** 4 ref

| **Field** | **Query** | **Hits** |
| --- | --- | --- |
| Abstract & Title | *ehealth *assessment | 4 |
| Subdiscipline | musculoskeletal |  |
| Match all search terms (AND) | |  |

**Website of the Swedish Agency for Health Technology Assessment and Assessment of Social Services**

**Date:** 1 June 2021
**No. of results:** 25 ref

| **Field** | **Query** | **Hits** |
| --- | --- | --- |
| N/A | digital | 25 |

**Google**

**Date:** 1 June 2021
**No. of results:** 100 ref

| **Field** | **Query** | **Hits** |
| --- | --- | --- |
| N/A | (digital OR tele OR ehealth OR mhealth OR app OR video OR online OR internet OR web based) AND (assessment OR examination OR evaluation OR test OR diagnosis) AND (”physical therapy” OR physiotherapy OR physiotherapist) AND musculoskeletal | 12,600,000 |
|  | Limited to scientific articles | 134,000 |
|  | Limited to last 4 years (2018-2021) | 16,900 |
|  | Limited to first 100 hits | 100 |

**Updated database searches May 25 2022**

**Database:** Ovid MEDLINE(R) ALL
**Date:** 2022-05-25
**No of results:** 178 ref

| **#** | **Query** | **Results from 25 May 2022** |
| --- | --- | --- |
| 1 | exp Musculoskeletal Diseases/ | 1,169,302 |
| 2 | limit 1 to yr="2021 -Current" | 50,613 |
| 3 | ((musculoskelet* or orthopedic* or ortopedic* or orthopaed* or ortopaed* or musc* or skelet* or bone* or cartilag* or joint* or shoulder* or elbow* or wrist* or neck* or cervical or back* or spine or spinal or lumb* or hip* or coxa* or knee* or ankle* or tars* or foot or feet) adj3 (disease* or condition* or disorder* or problem* or pain* or ache* or injur* or damage* or diagnos* or complaint* or dysfunction*)).ab,kf,ti. | 569,996 |
| 4 | limit 3 to yr="2021 -Current" | 62,728 |
| 5 | (backpain* or backache* or lumbago or dorsalgia or arthralgia* or polyarthralgia* or neckpain* or neckache* or cervicalgia* or cervicodynia*).ab,kf,ti. | 15,947 |
| 6 | limit 5 to yr="2021 -Current" | 1,277 |
| 7 | 2 or 4 or 6 | 102,549 |
| 8 | exp Telemedicine/ or exp Videoconferencing/ | 41,836 |
| 9 | limit 8 to yr="2021 -Current" | 6,984 |
| 10 | (assess* or examin* or measur* or evaluat* or test* or diagnos* or estimat* or judg* or visit* or consult* or counsel* or appointment*).ab,kf,ti. | 14,035,618 |
| 11 | limit 10 to yr="2021 -Current" | 1,329,650 |
| 12 | 9 and 11 | 4,923 |
| 13 | ((digital* or electronic* or virtual or interactive* or inter-activ* or tele* or ehealth* or e-health* or mhealth* or m-health* or app or apps or smartphone* or phone* or video* or online or internet* or webbased or web-based or computer*) adj6 (assess* or examin* or measur* or evaluat* or test* or diagnos* or estimat* or judg* or visit* or consult* or counsel* or appointment*)).ab,kf,ti. | 246,439 |
| 14 | limit 13 to yr="2021 -Current" | 37,256 |
| 15 | 12 or 14 | 39,145 |
| 16 | exp Physical Therapists/ or exp Physical Therapy Modalities/ | 172,858 |
| 17 | limit 16 to yr="2021 -Current" | 10,523 |
| 18 | (physical therap* or physiotherap* or physio-therap*).ab,kf,ti. | 58,580 |
| 19 | limit 18 to yr="2021 -Current" | 7,427 |
| 20 | 17 or 19 | 15,764 |
| 21 | 7 and 15 and 20 | 178 |

* ab = abstract, kf = author keyword, ti = title

___________________________________________________________________________

**Database:** The Cochrane Library
**Date:** 2022-05-25
**No of results:** 38 ref

*Cochrane reviews: 0
Cochrane protocols: 0
Trials: 38
Editorials: 0
Special collections: 0
Clinical answers: 0*

| **ID** | **Search** | **Hits** |
| --- | --- | --- |
| #1 | MeSH descriptor: [Musculoskeletal Diseases] explode all trees | 45176 |
| #2 | #1 with publication date within the last year | 2305 |
| #3 | ((musculoskelet* OR orthopedic* OR ortopedic* OR orthopaed* OR ortopaed* OR musc* OR skelet* OR bone* OR cartilag* OR joint* OR shoulder* OR elbow* OR wrist* OR neck* OR cervical OR back* OR spine OR spinal OR lumb* OR hip* OR coxa* OR knee* OR ankle* OR tars* OR foot OR feet) NEAR/3 (disease* OR condition* OR disorder* OR problem* OR pain* OR ache* OR injur* OR damage* OR diagnos* OR complaint* OR dysfunction*)):ti,ab,kw (with CL publication date within the last year, word variations have been searched) | 8696 |
| #4 | (backpain* OR backache* OR lumbago OR dorsalgia OR arthralgia* OR polyarthralgia* OR neckpain* OR neckache* OR cervicalgia* OR cervicodynia*):ti,ab,kw (with CL publication date within the last year, , word variations have been searched) | 660 |
| #5 | #2 OR #3 OR #4 | 10176 |
| #6 | MeSH descriptor: [Telemedicine] explode all trees | 3206 |
| #7 | #6 with publication date within the last year | 268 |
| #8 | MeSH descriptor: [Videoconferencing] explode all trees | 245 |
| #9 | #8 with publication date within the last year | 20 |
| #10 | #7 OR #9 | 281 |
| #11 | (assess* OR examin* OR measur* OR evaluat* OR test* OR diagnos* OR estimat* OR judg* OR visit* OR consult* OR counsel* OR appointment*):ti,ab,kw (with CL publication date within the last year, word variations have been searched) | 102017 |
| #12 | #10 AND #11 | 266 |
| #13 | ((digital* OR electronic* OR virtual OR interactive* OR "inter activ*" OR tele* OR ehealth* OR "e health*" OR mhealth* OR "m health*" OR app OR apps OR smartphone* OR phone* OR video* OR online OR internet* OR webbased OR "web based" OR computer* ) NEAR/6 (assess* OR examin* OR measur* OR evaluat* OR test* OR diagnos* OR estimat* OR judg* OR visit* OR consult* OR counsel* OR appointment*)):ti,ab,kw (with CL publication date within the last year, word variations have been searched) | 5950 |
| #14 | #12 OR #13 | 6081 |
| #15 | MeSH descriptor: [Physical Therapists] explode all trees | 153 |
| #16 | #15 with publication date within the last year | 19 |
| #17 | MeSH descriptor: [Physical Therapy Modalities] explode all trees | 29256 |
| #18 | #17 with publication date within the last year | 1695 |
| #19 | ("physical therap*" OR physiotherap* OR "physio therap*"):ti,ab,kw (with CL publication date within the last year, word variations have been searched) | 2026 |
| #20 | #16 OR #18 OR #19 | 3496 |
| #21 | #5 AND #14 AND #20 | 107 |
| #22 | (clinicaltrials or trialsearch):so | 34984 |
| #23 | #21 NOT #22 | 47 |
| #24 | (conference abstract):pt | 19120 |
| **#25** | **#23 NOT #24** | **38** |

* ab = abstract, kw = author keyword, pt = publication type, so = source, ti = title, CL = Cocrane Library

___________________________________________________________________________

**Database:** Cinahl (EBSCO)
**Date:** 2022-05-25
**Limiters:** Published Date: 20210501-; Exclude MEDLINE records **No of results:** 44 refFormulärets överkant

| **#** | **Query** | **Results** |
| --- | --- | --- |
| S13 | S4 AND S9 AND S12 | 44 |
| S12 | S10 OR S11 | 3,853 |
| S11 | AB ( physical therap* OR physiotherap* OR physio-therap* ) OR TI ( physical therap* OR physiotherap* OR physio-therap* ) | 3,845 |
| S10 | (MH "Physical Therapy Assessment") | 26 |
| S9 | S7 OR S8 | 6,790 |
| S8 | AB ( (digital* OR electronic* OR virtual OR interactive* OR inter-activ* OR tele* OR ehealth* OR e-health* OR mhealth* OR m-health* OR app OR apps OR smartphone* OR phone* OR video* OR online OR internet* OR webbased OR web-based OR computer*) N6 (assess* OR examin* OR measur* OR evaluat* OR test* OR diagnos* OR estimat* OR judg* OR visit* OR consult* OR counsel* OR appointment*) ) OR TI ( (digital* OR electronic* OR virtual OR interactive* OR inter-activ* OR tele* OR ehealth* OR e-health* OR mhealth* OR m-health* OR app OR apps OR smartphone* OR phone* OR video* OR online OR internet* OR webbased OR web-based OR computer*) N6 (assess* OR examin* OR measur* OR evaluat* OR test* OR diagnos* OR estimat* OR judg* OR visit* OR consult* OR counsel* OR appointment*) ) | 6,364 |
| S7 | S5 AND S6 | 954 |
| S6 | AB ( assess* OR examin* OR measur* OR evaluat* OR test* OR diagnos* OR estimat* OR judg* OR visit* OR consult* OR counsel* OR appointment* ) OR TI ( assess* OR examin* OR measur* OR evaluat* OR test* OR diagnos* OR estimat* OR judg* OR visit* OR consult* OR counsel* OR appointment* ) | 142,017 |
| S5 | (MH "Telemedicine+") OR (MH "Videoconferencing+") | 2,074 |
| S4 | S1 OR S2 OR S3 | 15,566 |
| S3 | AB ( backpain* OR backache* OR lumbago OR dorsalgia OR arthralgia* OR polyarthralgia* OR neckpain* OR neckache* OR cervicalgia* OR cervicodynia* ) OR TI ( backpain* OR backache* OR lumbago OR dorsalgia OR arthralgia* OR polyarthralgia* OR neckpain* OR neckache* OR cervicalgia* OR cervicodynia* ) | 167 |
| S2 | AB ( (musculoskelet* OR orthopedic* OR ortopedic* OR orthopaed* OR ortopaed* OR musc* OR skelet* OR bone* OR cartilag* OR joint* OR shoulder* OR elbow* OR wrist* OR neck* OR cervical OR back* OR spine OR spinal OR lumb* OR hip* OR coxa* OR knee* OR ankle* OR tars* OR foot OR feet) N3 (disease* OR condition* OR disorder* OR problem* OR pain* OR ache* OR injur* OR damage* OR diagnos* OR complaint* OR dysfunction*) ) OR TI ( (musculoskelet* OR orthopedic* OR ortopedic* OR orthopaed* OR ortopaed* OR musc* OR skelet* OR bone* OR cartilag* OR joint* OR shoulder* OR elbow* OR wrist* OR neck* OR cervical OR back* OR spine OR spinal OR lumb* OR hip* OR coxa* OR knee* OR ankle* OR tars* OR foot OR feet) N3 (disease* OR condition* OR disorder* OR problem* OR pain* OR ache* OR injur* OR damage* OR diagnos* OR complaint* OR dysfunction*) ) | 9,024 |
| S1 | (MH "Musculoskeletal Diseases+") | 8,838 |

* AB = abstract, MH = CINAHL subject heading, TI = titleFormulärets nederkant

___________________________________________________________________________

**Database:** AMED (EBSCO)
**Date:** 2022-05-25
**Limiters:** Published Date: 20210501- **No of results:** 0 ref

| **#** | **Query** | **Results** |
| --- | --- | --- |
| S6 | S3 AND S4 AND S5 | 0 |
| S5 | AB ( physical therap* OR physiotherap* OR physio-therap* ) OR TI ( physical therap* OR physiotherap* OR physio-therap* ) | 22 |
| S4 | AB ( (digital* OR electronic* OR virtual OR interactive* OR inter-activ* OR tele* OR ehealth* OR e-health* OR mhealth* OR m-health* OR app OR apps OR smartphone* OR phone* OR video* OR online OR internet* OR webbased OR web-based OR computer*) N6 (assess* OR examin* OR measur* OR evaluat* OR test* OR diagnos* OR estimat* OR judg* OR visit* OR consult* OR counsel* OR appointment*) ) OR TI ( (digital* OR electronic* OR virtual OR interactive* OR inter-activ* OR tele* OR ehealth* OR e-health* OR mhealth* OR m-health* OR app OR apps OR smartphone* OR phone* OR video* OR online OR internet* OR webbased OR web-based OR computer*) N6 (assess* OR examin* OR measur* OR evaluat* OR test* OR diagnos* OR estimat* OR judg* OR visit* OR consult* OR counsel* OR appointment*) ) | 14 |
| S3 | S1 OR S2 | 51 |
| S2 | AB ( backpain* OR backache* OR lumbago OR dorsalgia OR arthralgia* OR polyarthralgia* OR neckpain* OR neckache* OR cervicalgia* OR cervicodynia* ) OR TI ( backpain* OR backache* OR lumbago OR dorsalgia OR arthralgia* OR polyarthralgia* OR neckpain* OR neckache* OR cervicalgia* OR cervicodynia* ) | 1 |
| S1 | AB ( (musculoskelet* OR orthopedic* OR ortopedic* OR orthopaed* OR ortopaed* OR musc* OR skelet* OR bone* OR cartilag* OR joint* OR shoulder* OR elbow* OR wrist* OR neck* OR cervical OR back* OR spine OR spinal OR lumb* OR hip* OR coxa* OR knee* OR ankle* OR tars* OR foot OR feet) N3 (disease* OR condition* OR disorder* OR problem* OR pain* OR ache* OR injur* OR damage* OR diagnos* OR complaint* OR dysfunction*) ) OR TI ( (musculoskelet* OR orthopedic* OR ortopedic* OR orthopaed* OR ortopaed* OR musc* OR skelet* OR bone* OR cartilag* OR joint* OR shoulder* OR elbow* OR wrist* OR neck* OR cervical OR back* OR spine OR spinal OR lumb* OR hip* OR coxa* OR knee* OR ankle* OR tars* OR foot OR feet) N3 (disease* OR condition* OR disorder* OR problem* OR pain* OR ache* OR injur* OR damage* OR diagnos* OR complaint* OR dysfunction*) ) | 50 |

* AB = abstract, TI = title

___________________________________________________________________________

**Database:** PEDro
**Date:** 2022-05-25
**No of results:** 0 ref

| **Field** | **Query** | **Hits** |
| --- | --- | --- |
| Abstract & Title | *ehealth *assessment | 0 |
| Subdiscipline | musculoskeletal |  |
| Match all search terms (AND) | |  |
| New records added since 26-06-2021 | |  |
