# Supplementary material for: Digital physiotherapy assessment vs conventional face-to-face physiotherapy assessment of patients with musculoskeletal disorders: A systematic review
Source: PLoS One. 2023 Mar 21;18(3):e0283013. doi: 10.1371/journal.pone.0283013 (PMC10030027; doi:10.1371/journal.pone.0283013)
Supplement: S3 File — (DOCX) [file pone.0283013.s003.docx]

**Supporting file 3** List of excluded studies

| **Authors** | **Year** | **Title** | **Journal** | **Reason for exclusion** |
| --- | --- | --- | --- | --- |
| Galea M, Tumminia J, Garback LM. | 2006 | Telerehabilitation in spinal cord injury persons: a novel approach | [Telemed J e-Health: the official journal of the American Telemedicine Association. 12(2):160-2.](https://pubmed.ncbi.nlm.nih.gov/16620171/) | No digital assessment |
| Gilbert AW, Jaggi A, May CR. | 2019 | What is the acceptability of real time 1:1 videoconferencing between clinicians and patients for a follow-up consultation for multi-directional shoulder instability? | [Shoulder & Elbow. 11(1)53-9.](https://pubmed.ncbi.nlm.nih.gov/30719098/) | No digital assessment |
| Head J, James A, Sparrow P, Franklin N. | 2020 | Transforming musculoskeletal physiotherapy delivery to provide a combined digitalised service; a service evaluation | Physiother. P107. Supplement 1, e138, May 01, 202038, MAY 01, 2020 | Service evaluation |
| Hohenschurz- Schmidt D, Scott W, Park C, Christopoulos G, Vogel S, Draper-Rodi J. | 2020 | Remote management of musculoskeletal pain: a pragmatic approach to the implementation of video and phone consultations in musculoskeletal practice | [Pain reports. 5;5(6):e878.](https://pubmed.ncbi.nlm.nih.gov/33344873/) | Review |
| Coulter EH, McLean AN, Hasler JP, Allan DB, McFayden A, Paul L. | 2017 | The effectiveness and satisfaction of web-based physiotherapy in people with spinal cord injury: a pilot randomised controlled trial | [Spinal cord. 55(4):383-9.](https://pubmed.ncbi.nlm.nih.gov/27596027/) | No digital assessment |
| Albornoz-Cabello M, Barrios-Quinta CJ, Barrios-Quinta AM, Escobio-Prieto I, Cardera-Durán MdLA, Espejo-Antunez L. | 2021 | Effectiveness of Tele-Prescription of Therapeutic Physical Exercise in Patellofemoral Pain Syndrome during the COVID-19 Pandemic | [Int J Environ Res Public Health. 25;18(3).](https://pubmed.ncbi.nlm.nih.gov/33504042/) | No face-to-face comparator |
| Bearne LM, Gregory WJ, Hurley MV. | 2021 | Remotely delivered physiotherapy: can we capture the benefits beyond COVID-19? | Rheumatol (Oxford). 2021 Apr 6;60(4):1582-1584 | Editorial |
| Turner A | 2018 | Case Studies in Physical Therapy: Transitioning A "Hands-On" Approach into A Virtual Platform | Int J Telerehabil. 2018 Aug 3;10(1):37-50. | No face-to-face comparator |
| Werneke MW, Deutscher D, Grigsby D, Tucker CA, Mioduski JE, Hayes D | 2021 | Telerehabilitation During the Covid-19 Pandemic in Outpatient Rehabilitation Settings: A Descriptive Study | Phys Ther. 2021 Jul 1;101(7):pzab110. | No digital assessment |
| Wong B, Ward D, Gemmell K, Bright R, Blackman R, Sole G, Ward S | 2020 | How is telehealth being utilized in the context of rehabilitationfor lower limb musculoskeletal disorders: a scoping review | Phys Ther Rev. Volume 25 2020 Issue 5-6 Special Issue – Students Research Projects | Review |
| Tenforde AS, Hefner JE, Kodish-Wachs JE, Iaccarino MA, Paganoni S | 2017 | Telehealth in Physical Medicine and Rehabilitation: A Narrative Review | PM R. 2017 May;9(5S): S51-S58 | Review |
| Lovo S, Harrison L, O’Connell ME, Rotter T, Bath B | 2022 | A physical therapist and nurse practitioner model of care for chronic back pain using telehealth: Diagnostic and management concordance | J Telemed Telecare. 2022 May 12 | Wrong intervention |
| Mehta SB, Kendall KM, Reasor CM | 2021 | Virtual assessments of knee and wrist joint range motion have comparable reliability with face-to-face assessments | Musculoskelet Care. 2021 Jun;19(2):208-216 | Wrong population |
