# Supplementary material for: Digital physiotherapy assessment vs conventional face-to-face physiotherapy assessment of patients with musculoskeletal disorders: A systematic review
Source: PLoS One. 2023 Mar 21;18(3):e0283013. doi: 10.1371/journal.pone.0283013 (PMC10030027; doi:10.1371/journal.pone.0283013)
Supplement: S4 File — (DOCX) [file pone.0283013.s004.docx]

**Supporting file 4. Outcome tables**

**a) Clinical tests.**

| **Study** | **Patient population** | **Test** | **Agreement between digital and face-to-face** | | **Reliability** | | |
| --- | --- | --- | --- | --- | --- | --- | --- |
|  |  |  | **Validity** | **Measure** | **Inter-rater** | **Intra-rater** | **Measure** |
| Cabana 2010 (28) | Knee disorders | 30 s chair-to-stand test | 5% (-18% to 29%) 0.85 | % difference (95% CI) Krippendorff's alpha | NR | NR |  |
|  |  | Timed up and go (TUG) | -6% (-33% to 21%) 0.86 | % difference (95% CI) Krippendorff's alpha | NR | NR |  |
|  |  | Tinetti test | -2.5% (-25% to 20%) 0.79 | % difference (95% CI) Krippendorff's alpha | NR | NR |  |
|  |  | Berg balance | 1.7% (-10% to 13%) 0.76 | % difference (95% CI) Krippendorff's alpha | NR | NR |  |
|  |  | Joint swelling | 0% (-8% to 8%) 0.87 | % difference (95% CI) Krippendorff's alpha | NR | NR |  |
| Lade 2012 (22) | Elbow disorders | Special orthopaedic tests | 75%; p=0.003 | % agreement; p-value | 91%; p<0.001 | 94%; p<0.001 | % agreement; p-value |
|  |  | Self-resisted static muscle tests | 90%; p=0.006 | % agreement; p-value | 96%; p<0.001 | 98%; p<0.001 | % agreement; p-value |
|  |  | Nerve tests | 46%; p=0.616 | % agreement; p-value | 68%; p=0.003 | 98%; p<0.001 | % agreement; p-value |
|  |  | Joint assessment | 47%; p=0.386 | % agreement; p-value | 97%; p<0.001 | 81%; p<0.001 | % agreement; p-value |
|  |  | Limiting factor | 68%; p<0.001 | % agreement; p-value | 84%; p<0.001 | 86%; p<0.001 | % agreement; p-value |
| Mani 2021 (29) | Neck disorders | Endurance: Deep neck flexors | -2.28 (-4.46 to -0.11); (-8.25 to 3.68) | MD in seconds (95% CI); Bland Altman's limits of agreement | 0.99 (0.98 to 0.99) | 0.99 (0.98 to 0.99) | ICC (95% CI of ICC) |
| Palacin-Marin 2013 (30) | Back disorders | Endurance: Sorensen's test | 9.22 (-5.50 to 23.96);  0.80 | MD in seconds (95% CI); Cronbach's alpha | 0.92 (0.91 to 0.93) 0.93 | 0.94 (0.93 to 0.95) 0.95 | ICC (95% CI of ICC)  Cronbach's alpha |
|  |  | Functional assessment: ASLR | -0.66 (-0.39 to 0.26);  0.97 | MD on Likert scale (95% CI); Cronbach's alpha | 0.93 (0.92 to 0.94) 0.93 | 0.95 (0.94 to 0.96)  0.95 | ICC (95% CI of ICC) Cronbach's alpha |
| Richardson 2017 (23) | Knee disorders | Mixed clinical tests - categorical | 83%; 93%; 0.64 | % exact; %exact+similar agreement; weighted Kappa | 94%; 99%; 0.94 | 97%; 100%; 0.98 | % exact; %exact+similar agreement; weighted kappa |
|  |  | Mixed clinical tests - binary | 98.8%; 400.4; p<0.001 | % agreement; χ2; p-value | 99%; 982.4; p<0.001 | 100%; 1121.4; p<0.001 | % agreement; χ2; p-value |
| Russel 2010a (24) | Ankle disorders | Mixed clinical tests - categorical | 76.4%; 89.3%; 0.92 | % exact; %exact+similar agreement; weighted kappa | 90.8%; 97.3%; 0.98 | 94.3%; 99.2%; 0.99 | % exact; %exact+similar agreement; weighted kappa |
|  |  | Mixed clinical tests - binary | 99.3%; 234.41; p<0.001 | % agreement; χ2; p-value | 99.9%; 579.68; p<0.001 | 99.2%; 694.45; p<0.001 | % agreement; χ2; p-value |
| Russel 2010b (25) | Lower limb disorders | Mixed clinical tests - categorical | 77.3%; 90.3%; 0.76 | % exact; %exact+similar agreement; weighted kappa | 93.0%; 97.7%;  0.98 | 94.5%; 99.2%; 0.99 | % exact; %exact+similar agreement; weighted kappa |
|  |  | Mixed clinical tests - binary | 82.9%; 227.69; p<0.001 | % agreement; χ2; p-value | 95.1%; 827.23; p<0.001 | 97.4%; 969.81; p<0.001 | % agreement; χ2; p-value |
| Steele 2012 (26) | Shoulder disorders | Special orthopaedic tests | 75.9%; 54.77; p<0.001 | % agreement; χ2; p-value | 88.1%; 209.52; p<0.001 | 88.7%; 185.34; p<0.001 | % agreement; χ2; p-value |
|  |  | Nerve ROM and sensitisation | 56.1%; 6.29; p=0.012 | % agreement; χ2; p-value | 66.9%; 7.20; p=0.007 | 87.1%; 76.58; p<0.001 | % agreement; χ2; p-value |
|  |  | Strength | 87.1%; 31.55; p<0.001 | % agreement; χ2; p-value | 95.4%; 476.74; p<0.001 | 97.3%; 585.73; p<0.001 | % agreement; χ2; p-value |
|  |  | Joint assessment | 64.4%; 0.76; p=0.383 | % agreement; χ2; p-value | 90.5%; 43.99; p<0.001 | 85.9%; 51.00; p<0.001 | % agreement; χ2; p-value |
|  |  | Limiting factor | 68.1%; 320.18; p<0.001 | % agreement; χ2; p-value | 87.0%; 1549.90; p<0.001 | 88.9%; 1795.95; p<0.001 | % agreement; χ2; p-value |
| Truter 2014 (27) | Back disorders | SLR passive | 0.64; p<0.001 | Pearson's correlation | NR | NR |  |
|  |  | Agreement if painful | 90%; 28.59;  p<0.01 | % exact agreement; χ2; p-value | NR | NR |  |
|  |  | Agreement on symptoms | 84%; 0.62 | % exact agreement; kappa | NR | NR |  |
|  |  | Sensitivity dorsiflexion | 90%; 25.78;  p<0.01 | % exact agreement; χ2; p-value | NR | NR |  |
|  |  | Sensitivity hip internal rotation | 86%; 20.26;  p<0.01 | % exact agreement; χ2; p-value | NR | NR |  |
|  |  | Sensitivity neck active flexion | 82%; 2.17;  p=0.146 | % exact agreement; χ2; p-value | NR | NR |  |
| ASLR: Anterior straight leg raise; CI: Confidence interval; MD: Mean difference; NR: Not reported; SLR: Straight leg raise | | | | | |  |  |

**b) Range of motion.**

| **Study** | **Patient population** | **Test** | **Agreement between digital and face-to-face** | | **Reliability** | | |
| --- | --- | --- | --- | --- | --- | --- | --- |
|  |  |  | **Validity** | **Measure** | **Inter-rater** | **Intra-rater** | **Measure** |
| Cabana 2010 (28) | Knee disorders | ROM - flexion | -6% (-20% to 8%); 0.80 | % difference (95% CI) Krippendorff's alpha | N/A | N/A |  |
|  |  | ROM - extension | -6% (-20% to 6%); 0.85 | % difference (95% CI) Krippendorff's alpha | N/A | N/A |  |
| Lade 2012 (22) | Elbow disorders | ROM | 88%; p=0.001 | % agreement; p-value | 93%; p=0.000 | 95%; p<0.001 | % agreement; p-value |
| Mani 2021 (29) | Neck disorders | AROM - flexion | 1.20 (-0.18 to 2.58) | MD in centimeters (95% CI) | 0.98 (0.93 to 0.99) | 0.94 (0.98 to 0.99) | ICC (95%CI) |
|  |  | AROM - extension | -0.60 (-1.87 to 0.67) | MD in centimeters (95% CI) | 0.98 (0.92 to 0.99) | 0.97 (0.89 to 0.99) | ICC (95%CI) |
|  |  | AROM - rotation | -0.30 (-1.50 to 0.90) | MD in centimeters (95% CI) | 0.98 (0.95 to 0.99) | 0.99 (0.97 to 0.99) | ICC (95%CI) |
|  |  | AROM - side flexion | -1.00 (-2.53 to 0.53) | MD in centimeters (95% CI) | 0.96 (0.90 to 0.98) | 0.96 (0.90 to 0.99) | ICC (95%CI) |
| Palacin-Marin 2013 (30) | Back disorders | AROM - lateral flexion | 0.73 (-0.96 to 2.42); 0.751 | MD (95% CI); Cronbach's alpha | 0.92 (0.90 to 0.93) | 0.95 (0.94 to 0.96) | ICC (95%CI) |
|  |  | AROM - finger floor distance frontal | -0.5 (-1.88 to 0.87); 0.992 | MD (95% CI); Cronbach's alpha | 0.92 (0.91 to 0.93) | 0.94 (0.93 to 0.95) | ICC (95%CI) |
|  |  | AROM - finger floor distance lateral | 0.30 (-0.64 to 1.24);  0.972 | MD right side (95% CI); Cronbach's alpha | 0.93 (0.93 to 0.94) | 0.96 (0.95 to 0.96) | ICC (95%CI) |
| Steele 2012 (26) | Shoulder disorders | ROM | 87.4%; 30.78; p<0.001 | % agreement; χ2; p-value | 92.1%; 298.49; p<0.001 | 95.8%; 393.95; p<0.001 | % agreement; χ2; p-value |
| Truter 2014 (27) | Back disorders | AROM - flexion | 96%; 22.16; p<0.001 | % agreement; χ2; p-value | N/A | N/A |  |
|  |  | AROM - extension | 89%; 15.37; p<0.001 | % agreement; χ2; p-value | N/A | N/A |  |
|  |  | AROM - lateral flexion right | 81%; 10.40; p=0.001 | % agreement; χ2; p-value | N/A | N/A |  |
|  |  | AROM - lateral flexion left | 88%; 14.28; p<0.001 | % agreement; χ2; p-value | N/A | N/A |  |
|  |  | AROM - rotation right | 89%; 13.58; p<0.001 | % agreement; χ2; p-value | N/A | N/A |  |
|  |  | AROM - rotation left | 92%; 18.58; p<0.001 | % agreement; χ2; p-value | N/A | N/A |  |
| AROM: Active range of motion; CI: Confidence interval; CRE: Cronbach's reliability estimate; ICC: interclass correlation coefficient; MD: Mean difference; ROM: Range of motion | | | | | | | |

**c) Patient-reported outcome measures and pain assessment.**

| **Study** | **Patient population** | **Outcome** | **Agreement between digital and face-to-face** | | **Reliability** | | |
| --- | --- | --- | --- | --- | --- | --- | --- |
|  |  |  | **Validity** | **Measure** | **Inter-rater reliability** | **Intra-rater reliability** | **Measure** |
|  |  | **Pain** |  |  |  |  |  |
| Lade 2012 (22) | Elbow disorders | Change in pain during physical examination (Y/N) | 82%, p<0.001 | % agreement; p-value | 98%; p<0.001 | 97%; p<0.000 | % agreement; p-value |
| Mani 2021 (29) | Neck disorders | VAS (0-100) | 0.90 (95% CI -3.04 to 4.84); (-9.89 to 11.69) | MD (95% CI); Bland Altman's limits of agreement | 0.99 (0.97 to 0.99) | 0.99 (0.97 to 0.99) | ICC (95%CI) |
| Palacin-Marin 2013 (30) | Back disorders | VAS (0-100) | 1.06 (95% CI -4.49 to 6.62); 0.94 | MD (95% CI); Cronbach's alpha | NR | NR |  |
| Steele 2012 (26) | Shoulder disorders | Pain during physical examination (0-10) | 76.8%; 0.50 | % agreement; exact+similar weighted kappa | 97.2%; 0.95 | 97.2%; 0.95 | % agreement; exact+similar weighted kappa |
|  |  | Pain during physical examination (binary) | 81.7%; 70.867; p<0.001 | % agreement; χ2; p-value | 98.3%; 618.83; p<0.001 | 96.8%; 510.60; p<0.001 | % agreement; χ2; p-value |
|  |  | **Function/disability** |  |  |  |  |  |
| Mani 2021 (29) | Neck disorders | NPQ | 0.11 (-0.60 to 0.82) | MD (95% CI) | 1.00 (1.00 to 1.00) | 0.99 (1.00 to 1.00) | ICC (95% CI) |
| Palacin-Marin 2013 (30) | Back disorders | ODI | -0.26 (-0.97 to 0.44); 0.994 | MD (95% CI); Cronbach's alpha | NR | NR |  |
|  |  | **Health-related quality of life** |  |  |  |  |  |
| Palacin-Marin 2013 (30) | Back disorders | SF-12 Mental Component | -0.68 (-1.66 to 0.30); 0.973 | MD (95% CI); Cronbach's alpha | NR | NR |  |
|  |  | SF-12 Physical Component | 0.41 (-0.9 to 1.72);  0.971 | MD (95% CI); Cronbach's alpha | NR | NR |  |
|  |  | **Kinesiophobia** |  |  |  |  |  |
| Palacin-Marin 2013 (30) | Back disorders | TSK | -0.20 (-0.93 to 0.53); 0.977 | MD (95% CI); Cronbach's alpha | NR | NR |  |
| CI: Confidence Interval; MD: Mean difference; NPQ: Neck Pain Questionnaire; NR: Not reported; ODI: Oswestry Disability Index; SF-12: 12-Item Short Form Health Survey; TSK: Tampa Scale of Kinesiophobia; VAS: Visual Analogue Scale | | | | | | | |

**d) Posture.**

| **Study** | **Patient population** | **Test** | **Agreement between digital and face-to-face** | | **Reliability** | | |
| --- | --- | --- | --- | --- | --- | --- | --- |
|  |  |  | **Validity** | **Measure** | **Inter-rater** | **Intra-rater** | **Measure** |
| Mani 2020 (29) | Neck disorders | Sagittal head tilt angle | -0.96 (-1.45 to -0.47); 0.68; (-2.15 to 0.09) | MD in degrees (95% CI); SD; Bland Altman's limits of agreement | 0.97 (0.89 to 0.99); 1.04; 7.9%; 2.90 | 0.96 (0.83 to 0.99); 1.29; 9.0%; 3.59 | ICC (95% CI); SEM; CV; MDC |
|  |  | Craniocervikal angle | -0.88 (-1.49 to -0.27); 0.85; (-2.47 to 0.83) | MD in degrees (95% CI); SD; Bland Altman's limits of agreement | 0.93 (0.70 to 0.98); 3.41; 8.4%; 9.45 | 0.93 (0.69 to 0.98); 3.48; 8.1%; 9.65 | ICC (95% CI); SEM; CV; MDC |
|  |  | Shoulder angle | -0.32 (-0.89 to 0.25); 0.79; (-1.80 to 0.98) | MD in degrees (95% CI); SD; Bland Altman's limits of agreement | 0.99 (0.97 to 0.99); 1.57; 4.6%; 4.36 | 1.00 (0.98 to 0.99); 1.11; 5.0%; 3.09 | ICC (95% CI); SEM; CV; MDC |
| Truter 2014 (27) | Back disorders | Coronal symmetry | 56%; 0.43; p=0.51 | % exact agreement; χ2; p-value | NR | NR |  |
|  |  | Coronal scoliosis | 72%; 2.21; p=0.14 | % exact agreement; χ2; p-value | NR | NR |  |
|  |  | Coronal pelvic tilt | 52%; 0.17 | % exact agreement; Kappa | NR | NR |  |
|  |  | Coronal assymetry classification | 36%; 0.07 | % exact agreement; Kappa | NR | NR |  |
|  |  | Sagittal pelvic tilt | 75%; 0.10 | % exact agreement; Kappa | NR | NR |  |
|  |  | Sagittal pelvic position | 71%; nc | % exact agreement; Kappa | NR | NR |  |
|  |  | Lumbar lordosis | 25%; -0.20 | % exact agreement; Kappa | NR | NR |  |
|  |  | Thoracic kyphosis | 50%; 0.12 | % exact agreement; Kappa | NR | NR |  |
|  |  | Thoracic position | 67%; 0.19 | % exact agreement; Kappa | NR | NR |  |
| CI: Confidence interval; CV: Coefficient of variation; MD: Mean difference; MDC: Minimal detectable change; NR: Not reported; SD: Standard deviation; SEM: Standard error of measurement | | | | | | | |

**e) Patho-anatomical diagnosis and systems diagnosis.**

| **Study** | **Patient population** | **Outcome** | **Agreement between digital and face-to-face** | | **Reliability** | | |
| --- | --- | --- | --- | --- | --- | --- | --- |
|  |  |  | **Validity** | **Measure** | **Inter-rater reliability** | **Intra-rater reliability** | **Measure** |
| Cottrell 2018 (21) | Lumbar spine disorders | Patho-anatomical diagnosis | 42.9%; 50%; 92.9% | % exact; similar; exact+similar agreement | NR | NR |  |
|  | Shoulder disorders | Patho-anatomical diagnosis | 28.6%; 50%; 78.6% | % exact; similar; exact+similar agreement | NR | NR |  |
|  | Knee disorders | Patho-anatomical diagnosis | 42.9%; 35.7%; 78.6% | % exact; similar; exact+similar agreement | NR | NR |  |
| Lade 2012 (22) | Elbow disorders | Patho-anatomical diagnosis | 36%; 37%; 73% | % exact; similar; exact+similar agreement | 18%; 55%; 73% | 73%; 9%; 82% | % exact; similar; exact+similar agreement |
| Richardson 2017 (23) | Knee disorders | Patho-anatomical diagnosis | 67%; 22; 89% | % exact; similar; exact+similar agreement | 67%; 27%; 94% | 89%; 11%; 100% | % exact; similar; exact+similar agreement |
| Russel 2010a (24) | Ankle disorders | Patho-anatomical diagnosis | 53%; 40%; 93% | % exact; similar; exact+similar agreement | 46.7%; 53.3%; 100% | 93.3%; 6.7%; 100% | % exact; similar; exact+similar agreement |
| Russel 2010b (25) | Lower limb disorders | Patho-anatomical diagnosis | 68%; 11%; 79% | % exact; similar; exact+similar agreement | 63%; 26%; 89% | 84%; 16%; 100% | % exact; similar; exact+similar agreement |
| Steele 2012 (26) | Shoulder disorders | Patho-anatomical diagnosis | 18.5%; 40.7%; 59.3% | % exact; similar; exact+similar agreement | 23.1%; 50%; 73.1% | 40.7%; 59.3%; 100% | % exact; similar; exact+similar agreement |
| Lade 2012 (22) | Elbow disorders | Systems diagnosis | 73%, p=0.013 | % exact agreement; p-value | 64%; p=0.11 | 90%; p=0.001 | % exact aggreement; p-value |
| Richardson 2017 (23) | Knee disorders | Systems diagnosis | 94%; 12.5; p<0.001 | % exact agreement; χ2; p-value | 67%; 9.39; p=0.002 | 94%; 12.5; p<0.001 | % exact agreement; χ2; p-value |
| Russel 2010a (24) | Ankle disorders | Systems diagnosis | 80.0%; 4.27; p<0.04 | % exact agreement; χ2; p-value | 93.3%; 9.6; p<0.01 | 93.3%; 9.6; p<0.01 | % exact agreement; χ2; p-value |
| Russel 2010b (25) | Lower limb disorders | Systems diagnosis | 79%; 5.26; p=0.022 | % exact agreement; χ2; p-value | 89%; 3.91; p=0.048 | 100%; 13.46; p<0.001 | % exact agreement; χ2; p-value |
| Steele 2012 (26) | Shoulder disorders | Systems diagnosis | 78.6%; 35.70; p<0.001 | % exact agreement; χ2; p-value | 82.1%; 41.60; p<0.001 | 82.1%; 38.05; p<0.001 | % exact agreement; χ2; p-value |
| NR: Not reported |  |  |  |  |  |  |  |
