# Supplementary material for: Digital physiotherapy assessment vs conventional face-to-face physiotherapy assessment of patients with musculoskeletal disorders: A systematic review
Source: PLoS One. 2023 Mar 21;18(3):e0283013. doi: 10.1371/journal.pone.0283013 (PMC10030027; doi:10.1371/journal.pone.0283013)
Supplement: Attachment — Submitted filename: Response to reviewers.docx [file pone.0283013.s005.docx]

**Response to reviewers**

We thank the reviewers for their reviews and for the opportunity to improve our manuscript. We have addressed all comments raised and revised the manuscript when appropriate. Please see our point-to-point responses below, in *blue italic font*. Changes to the manuscript have been done using “tracked changes”.

**Journal Requirements:**

When submitting your revision, we need you to address these additional requirements.

1. Please ensure that your manuscript meets PLOS ONE's style requirements, including those for file naming. The PLOS ONE style templates can be found at

<https://journals.plos.org/plosone/s/file?id=wjVg/PLOSOne_formatting_sample_main_body.pdf> and

<https://journals.plos.org/plosone/s/file?id=ba62/PLOSOne_formatting_sample_title_authors_affiliations.pdf>

*We have reviewed the style templates and adjusted our affiliations accordingly.*

2. Please include a copy of Table 5 which you refer to in your text on page 17.

*Table 5 was submitted as a separate file and hence included at the very end of the manuscript, as it was too large to be included in the text according to journal requirements.*

**Review Comments to the Author**

**Reviewer #1:**

 1- Mani, S., Sharma, S., Omar, B., Paungmali, A., & Joseph, L. (2017). Validity and reliability of Internet-based physiotherapy assessment for musculoskeletal disorders: a systematic review. Journal of telemedicine and telecare, 23(3), 379-391. and
Grona, S. L., Bath, B., Busch, A., Rotter, T., Trask, C., & Harrison, E. (2018). Use of videoconferencing for physical therapy in people with musculoskeletal conditions: a systematic review. Journal of telemedicine and telecare, 24(5), 341-355.
You should explain the difference from studies. Especially your study is exactly the same as Suresh Manin's study. Even the studies you examine should clearly indicate your difference from that.

*Thank you for this comment. There are three main differences between our review and the two above-mentioned reviews:*

*- the literatures searches in those reviews were conducted in 2015 and 2016, respectively, whereas our literature search was conducted in 2021/2022. In view of the evolvement of digital health in recent years, as well as the COVID-19 pandemic, there was a need to perform a systematic review of the current literature.*

*- the previous reviews did not assess certainty of evidence in the findings, whereas we used the GRADE approach to assess certainty of evidence. This is a major strength of our systematic review in comparison to the previous reviews. We have added this clarification to the manuscript (page 30, line 649-650).*

*These differences, providing the rationale for our review, are stated on page six (lines 126-138).*

*-Lastly, our review includes three newer studies that were not included in the previous reviews. This is stated in the first paragraph in the discussion (page 24, line 498).*

2- It would be more appropriate to say physiotherapy instead of physiotherapist in the title.

*Thank you for this suggestion. We have changed the term to physiotherapy assessment, in the title and throughout the manuscript.*

3- Your method part is a bit long and complicated, you can simplify and edit it according to the article below.
Yagiz, G., Akaras, E., Kubis, H. P., & Owen, J. A. (2022). The Effects of Resistance Training on Architecture and Volume of the Upper Extremity Muscles: A Systematic Review of Randomized Controlled Trials and Meta-Analyses. Applied Sciences, 12(3), 1593.

*Thank you for this suggestion. We have shortened and simplified the methods section according to your suggestion (page 10-12).*

4- In the conclusion part, it should be emphasized in which evaluation parameters the remote evaluation is successful and in which it is unsuccessful. Make it clear that remote assessments are beneficial but not a substitute for face-to-face assessment.

*Thank you for this suggestion. We have added this to the conclusion (page 32-33).*

5- Reload Figure 1 with better resolution.

*We uploaded all figures in high resolution, but they are compressed by the editorial system for the peer review version. The resolution in the accepted version will be better.*

**Reviewer #2:**

Thank you for inviting me to review this article.

The authors did a good job. The manuscript was well-written, detailed and followed the PRISMA latest checklist for reporting systematic reviews. However, I have few observations to point out.

*Thank you, we appreciate your assessment!*

First, the reported location for "results of individual studies" item in the PRISMA 2020 checklist seems incorrect. Please check and make appropriate corrections.

*Thank you for discovering this error. We have corrected the location in the PRISMA checklist (S3).*

Second, the flow diagram is blurred, I could not read it. Can another diagram be provided?

*We uploaded all figures in high resolution, but they are compressed by the editorial system for the peer review version. The resolution in the accepted version will be better.*

Third, line 525, page 25: This sentence "...validity of the digital tests varies with the validity of the reference tests..." negates your definition of validity. Validity is the extent of agreement between both tests, hence the validity of one cannot vary form the other. Kindly rephrase the sentence appropriately.

*Thank you for this observation. The sentence was indeed open for misinterpretation, and we have removed it from the manuscript.*

Finally, do you think the search strategy developed by the subject librarian needed a review by another librarian before conducting the search?

*Thank you for this comment. The search strategy was developed by the author group, with support from a medical librarian and it was reviewed and revised several times during preliminary searches. We have clarified this in the manuscript (page 8, line 178). We do not believe it needed further review by another librarian.*
